# Investigation of Fatty Acid Ketohydrazone Modified Liposome's Properties as a Drug Carrier

**DOI:** 10.1155/2015/481670

**Published:** 2015-11-16

**Authors:** Keita Hayashi, Madoka Kiriishi, Keishi Suga, Yukihiro Okamoto, Hiroshi Umakoshi

**Affiliations:** ^1^Department of Chemical Engineering, National Institute of Technology, Nara College, 22 Yata-cho, Yamatokoriyama, Nara 639-1080, Japan; ^2^Division of Chemical Engineering, Graduate School of Engineering Science, Osaka University, 1-3 Machikaneyama-cho, Toyonaka, Osaka 560-8531, Japan

## Abstract

pH-responsive liposomes were prepared by modifying the liposome with acid-cleaving amphiphiles. Palmitic ketohydrazone (P-KH) or stearic ketohydrazone (S-KH), composed of hydrophilic sugar headgroup and hydrophobic acyl chain, was used as a modifier of the DMPC liposome. Because the ketohydrazone group of P-KH or S-KH was cleaved at low pH conditions (<pH 5.0), the delivery of the P-KH modified liposomes was observed probably via an endocytic pathway. The membrane properties of these liposomes were characterized, focusing on the variation of both polarity (measured by Laurdan) and membrane fluidity (measured by DPH) at low pH condition. The interface of the P-KH modified liposome at acidic pH was found to become more hydrophobic and less fluidic as compared with that at neutral pH; that is, P-KH modified liposome became more rigid structure. Therefore, it seems that the P-KH modified liposome could protect encapsulated drugs from the enzymes in the lysosome. This study shows the novel approach about design of pH-responsive liposomes based on the membrane properties.

## 1. Introduction

Various kinds of self-assembled aggregates, such as vesicle, micelle, and emulsion, are utilized as drug carrier in DDS (drug delivery system) research [1]. The self-assembled aggregates have previously been applied for the capsulation of the drug molecules since the 1980s [2, 3]. In a recent study, liposomes are regarded as one of the frequently used self-assembled aggregates in DDS application [3]. Drug-encapsulating liposomes have been already introduced in the medical treatment, for example, Myocet, a liposome encapsulating doxorubicin hydrochloride [4]. Although doxorubicin hydrochloride is treated as an antitumor drug, it is still known to induce strong side effects [5]. Therefore, more efficient encapsulation and targeting strategy are required to reduce side effects of doxorubicin hydrochloride by utilizing liposomes [4].

One of the most contributory factors to reduce side effects is the control of pharmacokinetics, especially, in the case of cancer chemotherapy [6]. Cancer cells are known to secrete some angiogenic growth factors, resulting in the recruitment of disorganized blood vessels to tumor. Disorganized blood vessels allow drug carrier (liposome, etc.) with ≅100 nm-diameter to invade to the tumor and to be delivered into the target cell [6]. On the other hand, blood vessels with mature walls prevent the delivery of drug carrier to other normal tissues [6]. The above inhibitory phenomenon has been reported as an enhanced permeability and retention (EPR) effect and, in general, the “*passive*” strategy to deliver the drugs into tumor cells should be selected by using the “stealth” type drug carrier (e.g., polyethylene glycol modified liposome) [4, 6].

As a post-step of the liposome delivery (approach of liposome into the plasma membrane), the delivery of the drug from liposome into the “cytosol” of the cancer cells is furthermore needed to improve the delivery efficiency [7]. Usually, anticancer drugs including doxorubicin hydrochloride show anticancer activity by binding to DNA of cancer cells [8]. The drug cannot be reaching the nuclei unless it could escape from the endosome inside the cytosol [7]. Various kinds of “*smart*” liposomes have previously been investigated for the escape of drug from endosomal pathway [9, 10]. The most usual one is pH stimulus-responsive liposome [9]. It is well known that the pH condition can be varied from neutral pH (~7) to acidic pH (~5) during the endosomal pathway through the contribution of the proton pump of ATPase bound on the membrane [11]. Among the possible smart materials that can make a response against the pH shift, a hydrazone bond is known as a pH-responsible functional group and its derivatives can be also utilized as the modifier of “*smart*” liposomes [12].

The key factor to design these liposomes is conventionally known as geometric structure of lipids. For example, phosphatidylethanolamine is frequently used as pH-responsive liposomes [13]. Phosphatidylethanolamine can release proton in low pH condition [14] and its headgroup can be reduced by the pH shift [15]. The above variation of the geometric structure at the headgroup of lipid can induce the membrane fusion with the endosomal membrane, resulting in the escape of encapsulated drug from endosome [16]. Although many researchers focus on this pH shift during the endocytotic pathway, there is no clear strategy for the carrier design, except for the above strategy based on the geometrical structure of carrier molecule.

In this study, the method to design the “membrane surface” of the pH-responsive liposome as “self-assembly” was investigated based on its physicochemical properties. The liposome membrane was modified by a pH-responsive amphiphilic molecule (palmitic ketohydrazone (P-KH) and stearic ketohydrazone (S-KH)) that has a ketohydrazone group in between glucose group (headgroup) and acyl chain region [17]. After the membrane properties of the liposome modified with P-KH or S-KH were characterized by using the previously reported method [18], the variation of the surface characteristics of the liposome was investigated under the pH shift. Based on the results on (i) entrapment of drug molecules in the liposome, (ii) its drug delivery efficiency, and (iii) its cytotoxicity, together with the physicochemical properties of the liposome at lower pH, a possibility to apply the P-KH liposome and S-KH liposome as drug carriers was finally investigated.

## 2. Materials and Methods

### 2.1. Materials

Phospholipid, 1,2-dimyristoyl-*sn*-glycero-3-phosphatidylcholine (DMPC), was purchased from NOF Corporation (Tokyo, Japan). Fluorescent probes, 1,6-diphenyl-1,3,5-hexatriene (DPH) and 6-dodecanoyl-N,N-dimethyl-2-naphthylamine (Laurdan), were purchased from Sigma Aldrich (St. Louis, MO, USA). d-Glucose was purchased from Kanto Chemical Co., Inc. (Tokyo, Japan). 2,4-Pentanedione was purchased from Wacker Chemie AG (München, Germany). Other chemicals were purchased from Wako Pure Chemical Industries, Ltd. (Osaka, Japan). Chemicals were used without further purification.

### 2.2. Synthesis of Palmitic Ketohydrazone (P-KH) and Stearic Ketohydrazone (S-KH)

**Figure 1:**
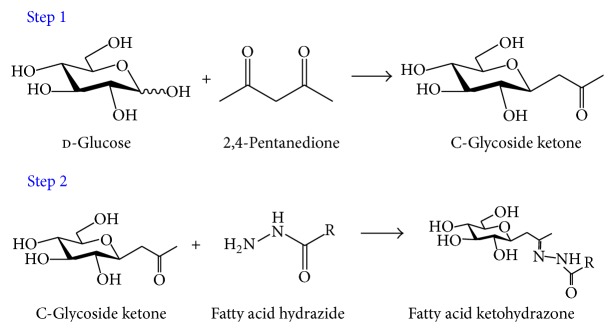
Scheme of fatty acid ketohydrazone synthesis.

Palmitic ketohydrazone (P-KH) and stearic ketohydrazone (S-KH) were synthesized by C-glycoside ketone and fatty acid hydrazide as shown in Figure 1 [17]. In order to synthesize C-glycoside ketone, 10 mmol of d-glucose, 10 mmol of sodium carbonate, and 12 mmol of 2,4-pentanedione were dissolved in 5 mL water. This solution was stirred for 4 hours at 90°C. Reacted solution was washed twice by ethyl acetate to remove the remaining 2,4-pentanedione from the reacted solution. After the aqueous phase was gently collected, C-glycoside ketone was lyophilized. 1.4 mmol of C-glycoside ketone and 2.1 mmol of fatty acid hydrazide were dissolved in 20 mL of methanol/ethanol (v/v = 1 : 1) and were stirred for 4 hours at 50°C. After removing the solvent by rotary evaporator, the excessive fatty acid hydrazide was removed by liquid-liquid extraction using water and 4-methyl-2-pentanone. Aqueous phase was gently collected, and the water was removed by freeze-drying completely. Obtained P-KH and S-KH were evaluated by mass spectroscopy, high pressure liquid chromatography, and FTIR to confirm the formation of hydrazone bond.

### 2.3. Preparation of P-KH Liposome and S-KH Liposome

P-KH liposome and S-KH liposome were prepared by thin film hydration method described previously [18]. 10~50 mol% of P-KH or S-KH was mixed with DMPC in chloroform/methanol. This organic solvent was removed by rotary evaporator. The residual lipid film, after drying under a vacuum overnight, was hydrated with PBS buffer (pH 7.3). The liposome suspension was subjected to five cycles of freezing and thawing and was then treated by extrusion device (Liposofast; Avestin Inc., Ottawa, ON, Canada) equipped with two layers of polycarbonate membranes with mean pore diameters of 100 nm.

### 2.4. Evaluation of Encapsulation Efficiency

Encapsulation efficiency of P-KH liposome was evaluated by using a model drug (calcein). P-KH liposome was prepared with 10 mM calcein solution. Nonencapsulated calcein was removed from P-KH liposome suspension by gel filtration (Sepharose 4B; GE Healthcare, Little Chalfont, UK). Concentration of DMPC was measured by choline oxidase/DAOS method (Phospholipids C Test Wako). Fluorescence intensity of calcein in the P-KH liposome was measured by fluorescence spectrophotometer FP-6500 (Jasco, Tokyo, Japan), after disruption of P-KH liposome by Triton X-100 in order to evaluate strictly encapsulation efficiency without effect of calcein self-quenching [19]. The number of entrapped calcein molecules in a liposome was calculated as follows:

(1) $$\begin{array}{c} \frac{number\;\;of\;\;calcein\;\;molecules}{liposome}=\frac{I_{\left(+\right)Triton}-I_{\left(-\right)Triton}}{C_{lipid}}, \end{array}$$

where *I*_(+)Triton_ and *I*_(−)Triton_ represent the fluorescence intensity of each sample after and before Triton X-100 treatment, respectively, and *C*_lipid_ is the total concentration of lipid.

### 2.5. Cell and Cell Culture

Murine rectal cancer cell line (Colon 26 cell) was obtained from RIKEN (RIKEN BRC Cell Bank, Ibaraki, Japan). Colon 26 cells were grown in Eagle's minimal essential medium (E-MEM) (Wako Pure Chemical Industries, Osaka, Japan) supplemented with 10% fetal bovine serum (FBS) (Thermo Fisher Scientific, Waltham, MA, USA). Colon 26 cells were calculated in the incubator (a humidified atmosphere consisting of 5% CO_2_ at 37°C).

### 2.6. Cytotoxicity Evaluation by MTT Assay

MTT assay was performed by using CellTiter 96 Nonradioactive Cell Proliferation Assay (Promega, Fitchburg, WI, USA) [20]. Colon 26 cells were seeded on the 96-well cell culture plate (100 *μ*L, 2.0 × 10^5^ cells/mL). These cells were cultured for 24 hours in E-MEM supplemented with 10% FBS in the incubator. A culture medium was exchanged by the new medium (100 *μ*L) containing liposomes (0–5 mM). The cells were incubated for 24 hours. 15 *μ*L of dye solution was added into the new medium. After incubation for 4 hours in the incubator, 100 *μ*L of solubilization solution/stop mix was added into the new medium. After incubation for 1 hour at 37°C, 5% CO_2_, an absorbance of each well was measured by microplate spectrophotometer, xMarkTM (Bio-Rad, Hercules, CA, USA).

### 2.7. Evaluation of Uptake Efficiency by Flow Cytometer and Confocal Laser Microscopy

Cellular uptake of P-KH liposome and S-KH liposome was evaluated by flow cytometry. Fluorescence labeled P-KH liposome and S-KH liposome were prepared by mixing with 0.5 mol% Rhodamine-PE (Avanti Polar Lipids, Alabaster, AL, USA). Cells were seeded on the 6-well cell culture plate (1.0 mL, 2.0 × 10^5^ cells/mL). Cells were cultured for 24 hours in E-MEM supplemented with 10% FBS in the incubator. A culture medium was exchanged by the new medium (1.0 mL) containing fluorescence labeled P-KH liposome and S-KH liposome (5.0 mM). After incubation for 12 hours, the cells were trypsinized and washed twice by PBS. Cells were analyzed by flow cytometer, Attune Acoustic Focusing Cytometer (Applied Biosystems, Inc. Foster City, CA, USA). After incubation for 4 hours, cells were observed by confocal laser microscopy, ECLIPSE TE 2000-U (Nikon, Tokyo, Japan).

### 2.8. Membrane Fluidity Measurement of DPH

The membrane fluidity of P-KH liposome and S-KH liposome was evaluated by measuring the fluorescence anisotropy of DPH (Ex: 360 nm, Em: 430 nm) in the liposomal membrane, using a fluorescence spectrophotometer FP-6500 (Jasco, Tokyo, Japan) with a polarizing plate. 10 *μ*L of 100 *μ*M DPH in ethanol was added into 1.0 mL of 0.25 mM P-KH liposome suspension, and the mixture was incubated for 30 min. Polarity (*P*) was calculated from the following equation:

(2) P=I0°0°−GI0°90°I0°0°+GI0°90°,G=I90°0°I90°90°,

where *I*_90°0°_ and *I*_90°90°_ are emission intensities perpendicular and parallel to the horizontally polarized light, respectively, and *G* is the correction factor. Since polarization is inversely proportional to fluidity, membrane fluidity is expressed as 1/*P*.

### 2.9. Characterization of Hydrophilic/Hydrophobic Membrane Properties by Laurdan

Hydrophilic/hydrophobic characterization of the vesicular membrane was evaluated by an environmentally sensitive fluorescence probe, Laurdan [21]. 1.0 *μ*L of 100 *μ*M Laurdan (in ethanol) was mixed with 1 mL of 100 *μ*M P-KH liposome or S-KH liposome suspension, and the mixture was incubated for more than 30 min. Laurdan emission spectra from 425 nm to 550 nm in the vesicles were obtained by using a fluorescence spectrophotometer FP-6500 (Jasco, Tokyo, Japan), when Laurdan was excited at 340 nm. Thus, the emission spectra were evaluated by calculation of *GP*_(340)_ value for each emission wavelength as follows:

(3) $$\begin{array}{c} {GP}_{\left(340\right)}=\frac{\left(I_{440}-I_{490}\right)}{\left(I_{440}+I_{490}\right)}, \end{array}$$

where *I*_440_ and *I*_490_ were fluorescence intensity of Laurdan at 440 nm and 490 nm, respectively. The values were obtained from the emission spectra using fixed exciting wavelengths of 340 nm. The temperature was kept at 37°C.

## 3. Results

### 3.1. Drug Encapsulation, Drug Delivery Efficiency, and Cytotoxicity of P-KH Liposome and S-KH Liposome

**Figure 2:**
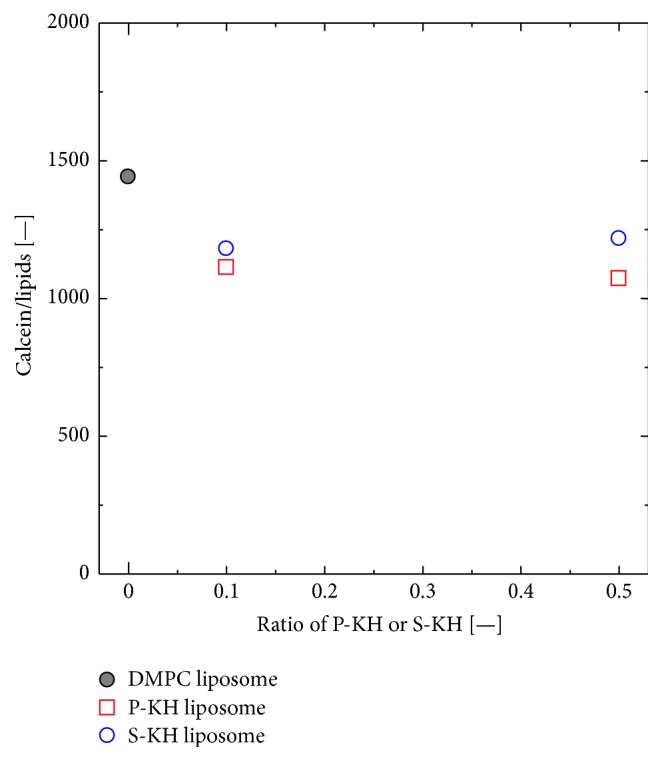
Entrapment of calcein in the liposomes. P-KH liposome and S-KH liposome could encapsulate equal amount of calcein as compared with DMPC liposome.

Entrapment of model drug (calcein) in P-KH liposome and S-KH liposome was evaluated by monitoring its fluorescence intensity. The number of calcein molecules per liposome was calculated as shown in Figure 2. The number of calcein molecules in the DMPC liposome was 1441 mol-calcein/mol-lipid, and those in the 10% P-KH liposome and 10% S-KH liposome were 1112 units and 1181 units, respectively. Although the number of calcein molecules in the P-KH liposome and S-KH liposome was slightly lower than that in DMPC liposome, P-KH liposome and S-KH liposome were found to encapsulate the calcein molecules inside their inner water pool. Moreover, the increase of the molar ratio of P-KH and S-KH hardly affected the numbers of calcein molecules in the liposomes, implying that the DMPC liposomes were successfully modified with P-KH and S-KH within the modifier ratio of 0–50%.

**Figure 3:**
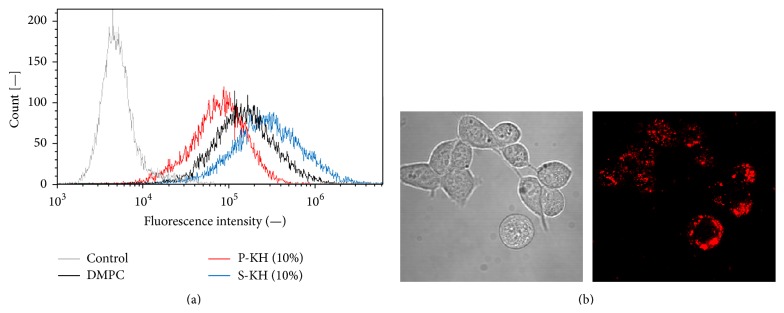
Drug delivery efficiency of P-KH liposome and S-KH liposome. (a) Quantitative evaluation of drug delivery efficiency by flow cytometer. (b) Images of confocal laser microscopy of colon 26 cells which were treated with fluorescence labeled P-KH liposome.

In order to evaluate the drug delivery efficiency, the uptake of three kinds of liposomes (such as DMPC liposome, P-KH liposome, and S-KH liposome) by colon 26 cells was evaluated by using flow cytometer (Figure 3(a)). This result shows that colon 26 cells took up similar amount of these liposomes. Therefore, both P-KH liposome and S-KH liposome can deliver the drug to the inside of cells and can also be applied as a drug carrier. Moreover, colon 26 cells were observed by confocal laser microscopy when colon 26 cells were treated with fluorescence labeled P-KH liposome (Figure 3(b)). Red fluorescence was observed at the inside of the cells. This result shows that colon 26 cells took up the P-KH liposome by endocytotic mechanism, judging from our previous report [20].

**Figure 4:**
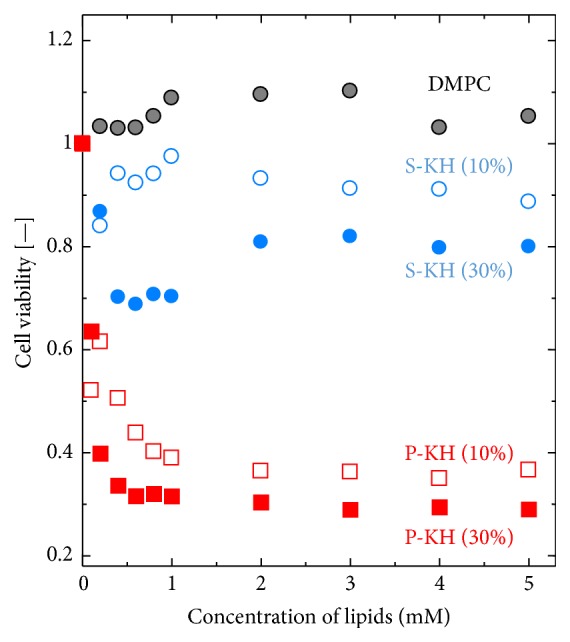
Cytotoxic effect of P-KH liposome and S-KH liposome on colon 26 cells. High concentration of P-KH liposome shows cytotoxicity effect on colon 26 cells. It is important to control a concentration of P-KH liposome in order to be used as a drug carrier.

The cytotoxicity of P-KH liposome and S-KH liposome is an important factor to apply them as a drug carrier. The cytotoxicity of the liposomes (DMPC liposome, P-KH liposome, and S-KH liposome) for colon 26 cells was evaluated by MTT assay.  Figure 4 shows the viability of colon 26 cells after their coincubation with DMPC liposome, P-KH liposome, or S-KH liposome. DMPC liposome and S-KH liposome hardly showed cytotoxic effect for colon 26 cells, although the cell viability of colon 26 cells was reduced to ~80% by the treatment with S-KH liposome. On the other hand, the viability of colon 26 cells was significantly reduced by P-KH liposome (10% and 30%) and reached less than 40%. It was thus investigated that only P-KH liposome showed cytotoxicity for colon 26 cells.

### 3.2. Variation of Membrane Properties of P-KH Liposome and S-KH Liposome at Lower pH Condition

It is well known that the proton pump of a membrane bound ATPase can transport proton across the endosomal membrane, suggesting that the P-KH liposome or S-KH liposome could be exposed to acidic pH in their use in the endosomal environment. In order to evaluate the pH effect on the membrane surface of P-KH liposome and S-KH liposome, the membrane properties of the liposomes were measured in lower pH conditions.

**Figure 5:**
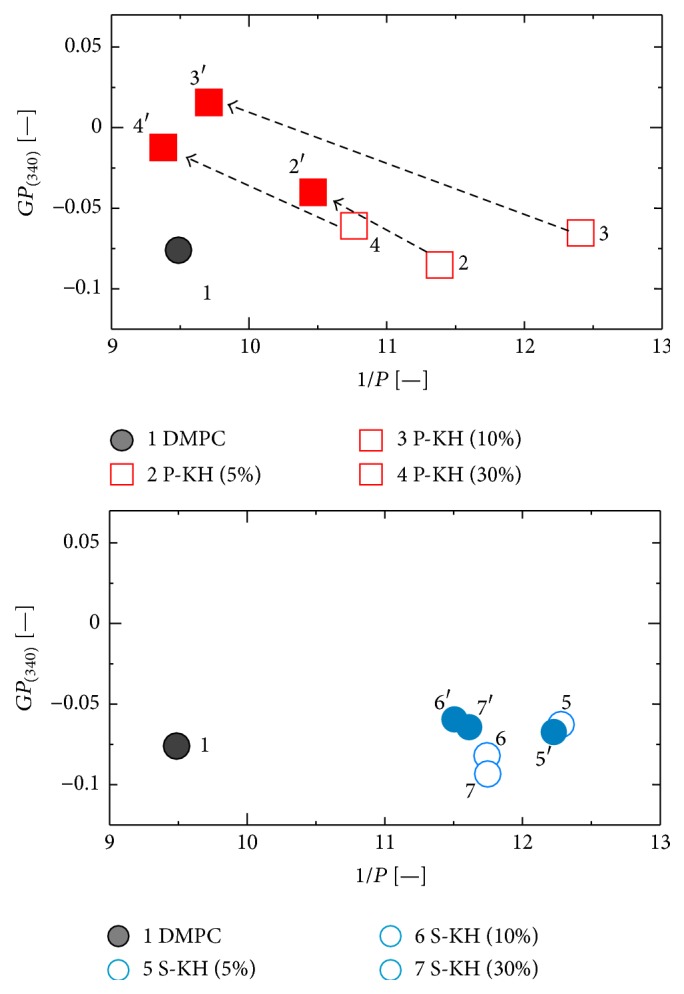
Alteration of membrane properties with dropping pH. Membrane properties were evaluated by membrane fluidity (1/*P*, anisotropy of DPH fluorescence) and hydrophilic/hydrophobic environment (*GP*_(340)_ values calculated by Laurdan fluorescence intensity) of liposomal membrane. 2~4 and 5~7 show the physicochemical properties of P-KH liposome and S-KH liposome in PBS, and 2′~4′ and 5′~7′ show that of P-KH liposome and S-KH liposome at pH 5.0.

Figure 5 shows pH dependence of the membrane fluidity (1/*P* value) and hydrophilic/hydrophobic environment (membrane polarity, *GP*_(340)_ value) of the liposomes. The 1/*P* values of P-KH liposomes decreased at pH 5, while the *GP*_(340)_ values of P-KH liposomes increased at pH 5. It has been reported that a hydrazone bond is stable at pH ~7, and it turns out to be cleaved at lower pH less than 5 [22]. Therefore, P-KH was assumed to be cleaved to C-glycoside ketone and palmitic acid hydrazide at pH 5, where the P-KH molecules on the liposomes can be partly altered to palmitic acid. The variation of the P-KH composition on the liposome membrane could induce the alteration of the membrane properties during the pH shift. This type of pH shift is, in general, observed inside the cell during its endocytotic pathway. Although the membrane properties of S-KH liposome did not show significant alteration by pH gradient, it is notable that the membrane fluidities of liposomes increased by modification both with P-KH and with S-KH.

## 4. Discussion

In this study, the P-KH liposome and S-KH liposome were prepared as pH-responsive drug carrier and their membrane properties were analyzed by using fluorescent probes. In recent years, various kinds of stimulus-responsive drug carrier were investigated in order to deliver drug effectively for diseased cells [9, 10]. A pH gradient is one of the most available stimulations for controlled release of drugs [9]. Some kinds of pH-responsive drug carriers have been previously investigated [7]. Here, P-KH liposomes and S-KH liposomes were characterized as the pH-responsive drug carriers, and the membrane properties, such as membrane fluidity (1/*P*) and membrane polarity (*GP*_(340)_), were investigated at lower pH condition that can imitate the endosomal condition. The membrane properties of the liposome could be related to its feature as a drug carrier; for example, the membrane fluidity of liposome significantly relates to the drug release from the liposomal inner phase [19]. When the pH value of P-KH liposome suspension can alter from pH 7 to pH 5, the 1/*P* values of P-KH liposomes decreased and the *GP*_(340)_ values increased, indicating that the P-KH liposome membranes turned out to be rigid at pH 5. These characteristics of P-KH liposomes will contribute to predicting the behaviors of P-KH liposome in the endosome.

However, S-KH liposomes indicated no clear variation of membrane properties by pH gradient, though the chemical structure of S-KH is very similar to that of P-KH. In general, the phase transition temperature of DSPC liposome, composed of “*stearic acid*,” from L_*β*_ phase to P_*β*_ phase is known to be 49.1°C and that from P_*β*_ phase to L_*α*_ phase is 54.5°C [23]. In addition, the value of DPPC liposome, composed of “*palmitic acid*,” from L_*β*_ phase to P_*β*_ phase is 34.4°C and that from P_*β*_ phase to L_*α*_ phase is 41.3°C [23]. The phase transition temperature of “*stearic acid-based (S-based)*” lipid seems to be higher than that of “*palmitic acid-based (P-based)*” lipid, implying the “*relatively rigid*” nature of “*S-based*” lipid. It is therefore suggested that the S-KH and P-KH could also show different behaviors at the interior of the membrane of “self-assembly (liposome).” The phase transition temperature of DMPC liposome is known to be 23.6°C [23], so that the phase separation of “*relatively rigid*” S-KH could occur on the “*relatively fluid*” DMPC liposome membrane at 37°C. The region of “*relatively rigid*” S-KH phase on the DMPC liposome membrane is not easily affected by pH gradation as compared with P-KH owing to the less flexibility of lipid molecules around the “*relatively rigid*” region. Therefore, P-KH liposomes turned out to be rigid, while S-KH liposomes showed higher membrane fluidities as compared to DMPC liposome.

It seems that the variation of the membrane properties of P-KH liposomes could be caused by the cleavage of hydrazone bond that conjugating with headgroup (glucose-glycoside ketone) and hydrophobic tail (palmitic acid hydrazide). The reduction of pH altered the partial amount of P-KH molecules to palmitic acid hydrazide on the liposome surface. It has been reported that the composition of vesicle including liposome directly affects the membrane properties of vesicles [24]. Han has previously reported that the diffusion coefficient of fatty acid was different from that of phospholipid on the lipid bilayer membrane by molecular dynamic simulation, in spite of same acyl chain structure between fatty acid and phospholipid [25]. Focusing on the membrane-membrane interaction, a higher headgroup mobility of Span 80 vesicle could be a possible key to induce the perturbation of target membrane [24]. It seems that the headgroup structure was important factor to control the membrane properties and functions. Therefore, P-KH liposome can be used as a smart (pH variable) drug carrier based on membrane properties by pH stimulation.

In the case of possible application of P-KH liposome in actual DDS, the cytotoxicity of P-KH liposome could also be a potential problem. In this study, P-KH liposome at the higher concentration shows the cytotoxicity for colon 26 cells (Figure 4). The results imply that there could be a possible problem of applicability of its P-KH liposome as a gene carrier although further investigation is needed to explain the cytotoxicity of P-KH liposome not only* in vitro* but also* in vivo*. The above findings also show that the P-KH liposome itself has a potential to function as an antitumor agent. It seems that the “smart” characteristics of the P-KH liposome surface could be utilized for its use against a solid tumor, considering the EPR effect [26].
